# Effect of scapular posterior tilting exercise on scapular muscle activities in men and women with a rounded shoulder posture

**DOI:** 10.1186/s13018-024-04810-z

**Published:** 2024-06-28

**Authors:** Qian Gu, Longlu Pan, Lijun Yu, Qin Jiang

**Affiliations:** 1 Department of Rehabilitation, Affiliated Hospital of Nantong University, No. 20. Xisi Road, Nantong, 226001 China; 2 The Rehabilitation Department of Affiliated Nantong Hospital of Shanghai University (The Rehabilitation Department of Sixth People’s Hospital of Nantong), Nantong, 226011 China; 3 Department of Clinical Medicine, Suzhou Vocational Health College, Suzhou, 215009 China; 4 Outpatient Department, Affiliated Hospital of Nantong University, No. 20. Xisi Road, Nantong, 226001 China

**Keywords:** Round shoulder posture, Scapular muscle, Scapular posterior tilt exercise, Electromyographic signal

## Abstract

Round-shoulder posture (RSP) is a common postural condition, characterized by protraction, downward rotation, anterior tilting and internal rotation of the scapula. RSP can lead to shoulder dysfunction. Different methods have been proposed for rehabilitating and correcting the altered posture in RSP including stretching, strengthening exercises, and shoulder brace or taping. However, the findings are controversial and studies are ongoing to develop more effective method. The present study is aimed at investigating the effects of scapular posterior tilting (SPT) exercise in different support positions on scapular muscle activities in men and women with RSP. In a prospective observational clinical study, we assessed demographic, basic clinical parameters and study variables of the subjects with RSP (n = 20) (men/women = 9/11) attending Daegu University in Gyeongsan, South Korea. To do so, we compared electromyographic (EMG) activities of lower trapezius and serratus anterior muscles between men and women with RSP during SPT exercise on four different support surfaces to determine any difference in the EMG activities. The results revealed that women showed significant differences in EMG activities in the lower and left upper trapezius and serratus anterior muscles, while men showed significant differences in EMG activity only in the lower trapezius muscle during SPT exercise on four different surfaces (*P* < 0.05). The post-hoc analysis revealed significantly greater EMG activity values in the lower trapezius and serratus anterior muscles during SPT exercise on the upper body unstable surface and whole-body unstable surface (*p* < 0.05). Independent t-tests after the Bonferroni correction showed no significant differences in muscle activities between men and women on the four different surfaces (*p* > 0.0125).

## Introduction

Rounded-shoulder posture (RSP) is one of the most common structural anomalies of the shoulder complex that is significantly associated with shoulder dysfunction [1, 2]. This altered postural condition is characterized by a protracted, anteriorly tipped and downwardly rotated of scapula position with elevated cervical lordosis and kyphosis of upper thoracic [3–5]. Etiologies of RSP include loss of lower trapezius (LT) and serratus anterior (SA) activity, tightness in the pectoralis minor (PM), elevated thoracic kyphosis, as well as the anatomical structure of the scapular itself. Abnormal scapular kinematics and the resulting imbalance in muscle in RSP make the anterior acromion transform into a close proximity to the supraspinatus and infraspinatus tendons which in turn increases the risk for subacromial impingement [6]. In the patient with RSP, the serratus anterior and lower trapezius positions that reportedly affect scapular, incline negatively [7, 8]. These alterations and transforms elevate tension and stress of muscle in the neck and shoulder resulting in numbness, discomfort, dysfunction or lack of function, and several neuromuscular symptoms and these alterations mostly affect the upper body of the patient [9].

Evidence shows that excessive antagonistic muscle activation during movements could reduce motor efficiency, which needs a coactivation compensation process.

The absence of coactivation might lead to joint instability and a detrimental phenomenon to motor control [10]. During this process, different individual factors play roles, of them, gender is important and worth further investigation from clinical and biomechanical aspects. At the first level, epidemiological studies have demonstrated that shoulder pain is more frequent in women than men [11, 12]. This higher risk of shoulder pain in women have been attributed to different anthropometrical, biomechanical and motor control-related differences that could affect motor behaviors [13, 14]. However, gender differences in strength, might contribute to the sex imbalance in the prevalence of musculoskeletal disorders more than in coactivation patterns [15].

Different initial studies have introduced several rehabilitation techniques for RSP including strengthening exercises for serratus anterior and lower trapezius muscles, correcting [16–19]. Strengthening lower trapezius and serratus anterior muscles are traditionally employed in rehabilitation program to compensate for the RSP induced decreased strength and mobility [20, 21]. Among the different exercises, scapular posterior tilt (SPT) exercise in the prone position and stabilizing the scapular to the thoracic wall have been reportedly the most efficient tools for strengthening serratus anterior and lower trapezius muscles [22]. Previous studies have demonstrated that using an unstable base of support during prone position training could further recruit neuromuscular system and improve muscle activation for shoulder rehabilitation [23]. However, the findings are controversial and further studies are needed to shed more light on the effective parameters on the effectiveness of the rehabilitation method to develop more effective method. The present study aimed to investigate the effects of SPT exercise in different support positions on scapular muscle activities in men and women with RSP.

## Methods

### Experimental subjects

This prospective observational clinical study was conducted on 20 subjects (men: 9; women: 11) diagnosed with RSP attending Daegu University in Gyeongsan, South Korea. The demographic information and basic clinical and experimental parameters and EMGs of the subjects were recorded (Table 1). All subjects were instructed to perform SPT exercise on four different support surfaces and during the exercises, the EMG activities were recorded in the right upper and lower trapezius muscles and in the opposite upper trapezius and serratus anterior muscles in all subjects.

**Table 1:** Demographic information and general clinical data of all subjects of the study

Variable	Men (n = 9) (M ± SD)	Women (n = 11) (M ± SD)	t (*p*)
Age	24.33 ± 1.87	24.45 ± 2.81	-0.111 (.082)
Height (cm)	175.78 ± 5.36	163.27 ± 4.13	5.904 (.504)
Weight (kg)	76.00 ± 8.90	55.94 ± 4.74	6.462 (.064)
BMI	24.56 ± 2.34	21.00 ± 1.77	3.983 (.986)
RSP distance (cm)	7.90 ± 0.96	5.56 ± 0.93	5.512 (.983)

*p < .05; Mean ± SD: Mean ± Standard Deviation

All experimental procedures of this study were approved by the local Institutional Review Board of Daegu University in Gyeongsan, South Korea (IRB code: 1,040,621–202203-HR-029) that completely coincide with the ethical standards and regulations of the studies on human beings set by the Helsinki declaration (2014)) [31]. The purpose of the study and potential benefits and possible risks of the experiments were clearly explained to all patients and they were instructed how to perform the exercises. Written informed consent forms for participating into the study were obtained from all patients. The inclusion criteria were the age range of 18–65 years old and RSP distance of ≥ 2.5 cm [24]. The exclusion criteria were presence of any activity-limiting injury or cervical fracture, history of shoulder disease.

### Experimental procedure

Twenty patients with RSP completed the study. They performed SPT exercise on four different surfaces. In all experiments, the subjects were instructed to perform the tasks with their dominant arm (favored arm for eating and writing tasks) [20]. Each patient repeated the exercise three times and the averaged variables were used for further analyses. All subjects stated that their dominant arm was the right arm.

### Experimental assessment

**Fig. 1:**
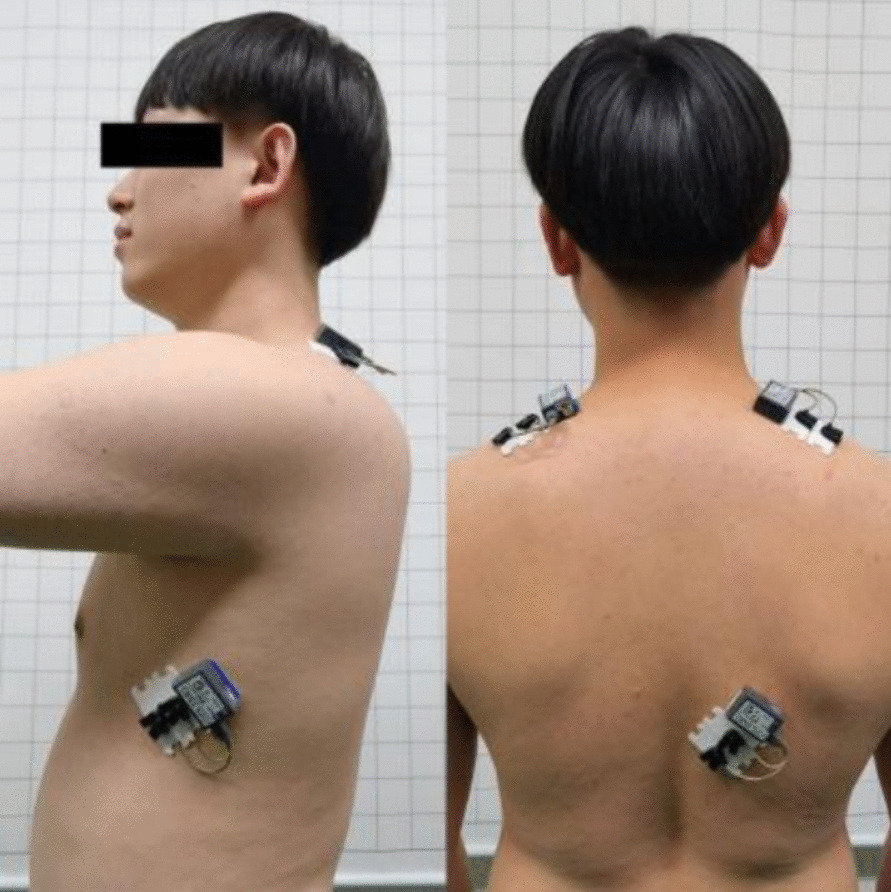
The placement of the surface electrodes for recording electromyographic signals

The distance from the posterior side of the acromion to the table was measured in the supine position using a straight ruler, and the abduction angle of the shoulder joint during the SPT exercise performance was measured with an angle-measuring instrument. The angle-measuring instrument refers to the Angle measuring system in wavelength dispersive X-ray fluorescence spectroscopy. It uses a rotating arm drive mechanism to measure the Angle. In this study, the cross-side horizontal Angle measurement method was adopted: the subject was in the standing or sitting position, and the upper limb was relaxed and naturally perpendicular to the body. The tester held the Angle measuring instrument or ruler, put one arm on the measuring instrument or ruler, and extended the shoulder joint to the maximum extent. Then the reading recorded the shoulder joint abduction Angle. The same standing position and EMG electrodes placements for normalizing the EMG signals were used in all subjects (Fig. 1). Before recording the study variables and at the start of the experiments, the normalization and standardization of EMG recordings to record the artefact- and bias-free EMG signals according to the previously published method [25]. This procedure was as follows: The subject was asked to perform maximal voluntary isometric contraction (MVIC) against manual resistance in the right lower trapezius and upper trapezius muscles then in the opposite serratus anterior and upper trapezius muscles [25]. The MVIC provides the reference of electrical activity for each muscle. All EMG data were presented as a percentage of MVIC. The process for each muscle was performed as follows: Lower trapezius muscle: In the all-fours position, the subject lifted the arm over the head in line with the lower trapezius muscle fibers while applying resistance above the elbow; Serratus anterior muscle—In the supine position, the subject performed the scapular protrudes at 90° of shoulder flexion when resistance was applied across the hand and at the elbow; Upper trapezius muscle: sitting in the erect position with no back support, the scapula was lifted with the neck-first side bent to the same side, rotated to the other side, and then extended, while resistance was applied at the head and above the elbow [8]. Between each contraction, a 2-min rest interval was permitted.

The MVIC value was calculated as follows: The root-mean-square (RMS) values were collected for the 3 s in the center, eliminating 1-s length of the signal from the beginning and end of the five sec length measures, and the average of the middle three values was calculated and recorded. Each MVIC was recorded as a 5-s length signal for each of the 2 trials.

### Experimental intervention

**Fig. 2:**
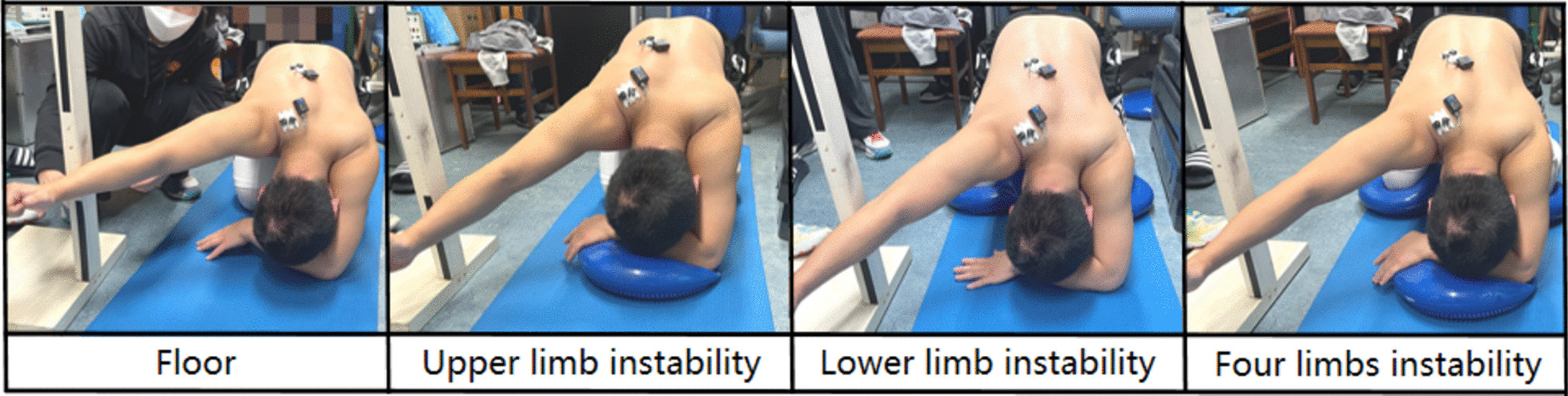
SPT exercise performed on four different support surfaces

In all subjects, the SPT exercise was performed on four different support surfaces (Fig. 2).

Floor: On the ground, the subject in the quadruped position was asked to rock slowly backward until achieving contact between the buttocks and both heels [22]. The subject was then instructed to lift the experimental arm until it reached the radial boundary of the wrist near the ear.

*Upper limb instability* Other conditions were the same as the Floor, except that an aircushion balance ball was placed under the non-experimental arm.

*Lower limb instability* Other conditions were the same as the Floor, except that four aircushion balance balls were placed under both sides of the lower.

*Four limps (whole body) instability* The combination of upper limb instability and lower limb instability.

### Statistical analysis

Statistical analyses of this study were performed with Statistical Package for Social Sciences (SPSS) ((IBM SPSS Statistics Inc., Chicago IL, Windows version 26.0). The normal distribution of all the continuous variables was evaluated with Kolmogorov–Smirnov normality test. The variables had normal distribution and were presented as mean ± standard deviation (M ± SD). One-way ANOVA was used to assess differences in the muscle activity and muscle activity ratios of both sides during SPT exercise performed on different surfaces in the RSP subjects. Independent t-tests were used to assess differences in muscles activities between men and women during exercise on four different surfaces. To reduce the Type I error, we used the Bonferroni correction. Bonferroni-corrected *P*-value was defined as $\frac{significant level}{n}$, where n denotes the number of surfaces in the exercise (n = 4). Bonferroni-corrected *P*-value = $\frac{0.05}{4}$, so the Bonferroni adjustment was set to *p* < 0.0125 for statistical significance.

## Results

### General subjects’ characteristics

Twenty subjects (men: 9; women: 11) finished the study. General characteristics of these subjects are represented in Table 1.

### Changes in EMG activities between four different support surfaces in men and women

**Fig. 3:**
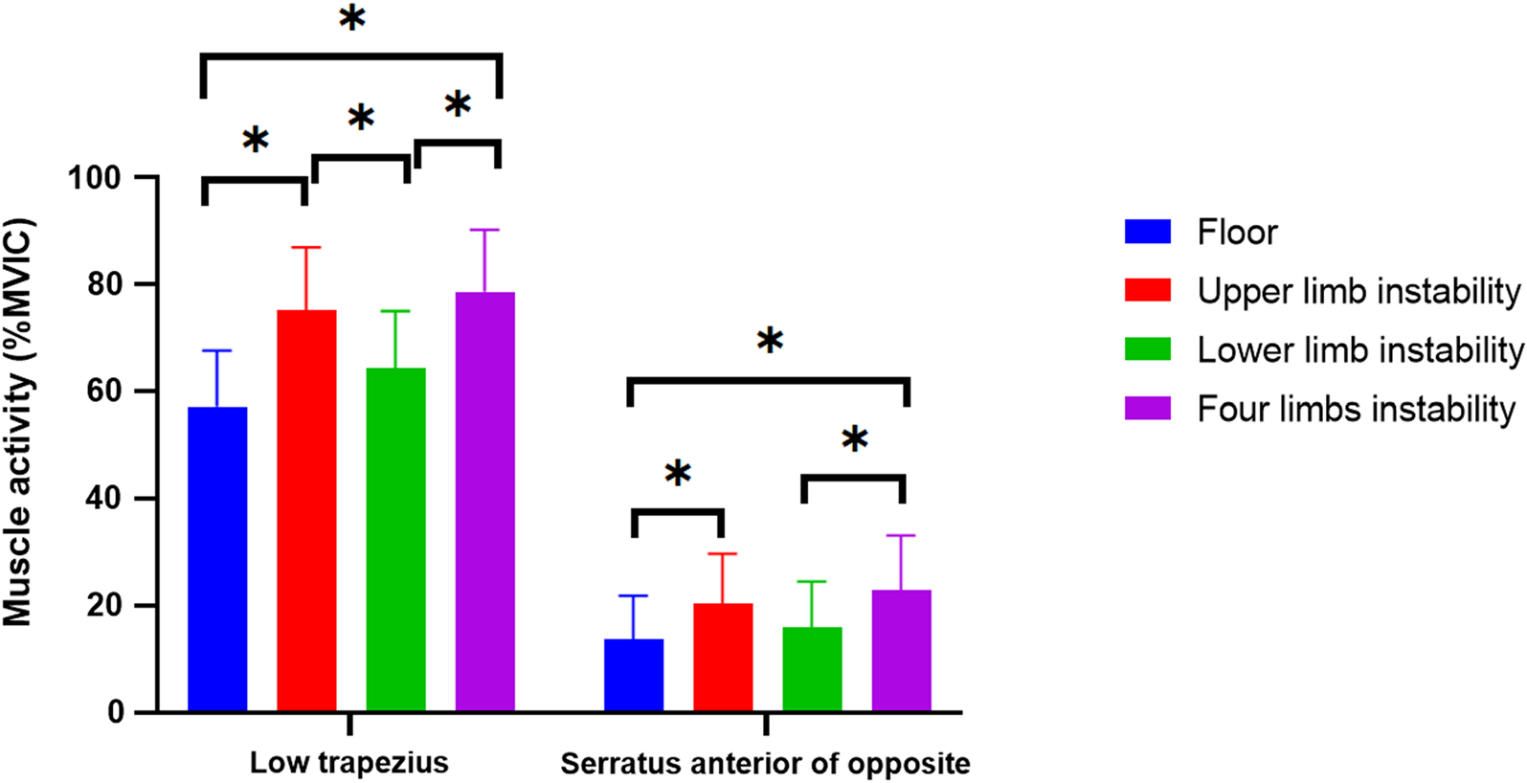
Comparison of muscle activities among four different support surfaces

The one-way ANOVA tests showed that concerning the lower trapezius muscle, both men and women showed significant differences (*p* < 0.05), and concerning the opposite upper trapezius muscle and serratus anterior muscle, only women showed significant differences (*P* < 0.05; Table 2). There were no significant variations in muscle activity between men and women among the four different surfaces, respectively (*P* > 0.0125; Table 2). Concerning the lower trapezius muscle, men and women showed the same results, where EMG activity was significantly increased on the upper body and whole-body unstable surfaces (*p* < 0.05), while in the opposite upper trapezius muscle and serratus anterior muscle, only women showed significantly increased EMG activities on the whole-body unstable surface (*p* < 0.05; Table 2). The results of the Bonferroni post hoc analysis showed that the EMG activities of the lower trapezius muscle and opposite serratus anterior muscle were significantly increased during SPT exercise on the whole-body unstable surface and upper body unstable surface (*p* < 0.05; Fig. 3).

**Table 2:** Comparisons of muscle activities in the subjects (men and women) on different surfaces N = 20 (Unit:%MVIC)

		A (M ± SD)	B (M ± SD)	C (M ± SD)	D (M ± SD)	F	*p*
LT	Men	63.21 ± 8.67	81.02 ± 10.69^a^	69.46 ± 10.21^b^	83.91 ± 10.95^a^	8.238	0.000*
	Women	52.40 ± 9.30	70.43 ± 10.67^a^	59.99 ± 9.60^b^	74.36 ± 10.53^a^	10.868	0.000*
	t	2.665	2.206	2.132	1.984		
	P’	0.016	0.041	0.047	0.063		
UT	Men	29.81 ± 6.68	34.88 ± 9.63	32.74 ± 8.73	37.61 ± 11.32	1.152	0.343
	Women	27.25 ± 10.49	32.86 ± 12.20	29.93 ± 11.03	34.11 ± 12.19	0.785	0.509
	t	0.631	0.404	0.620	0.660		
	P’	0.536	0.691	0.543	0.518		
SAO	Men	15.03 ± 10.59	21.07 ± 12.27	17.12 ± 11.21	23.32 ± 12.73	0.919	0.443
	Women	12.86 ± 5.66	19.76 ± 7.01	15.31 ± 5.75	22.75 ± 8.28^a^	4.715	0.007*
	t	0.586	0.300	0.466	0.121		
	P’	0.565	0.768	.647	.905		
UTO	Men	12.42 ± 9.28	16.23 ± 9.87	14.03 ± 9.36	15.06 ± 7.04	0.293	0.830
	Women	9.85 ± 3.23	13.63 ± 4.53	11.87 ± 3.82	15.65 ± 5.20^a^	3.715	0.019*
	t	0.793	0.081	0.088	0.477		
	P’	0.447	0.443	0.493	0.829		

*p < .05; **p’ < .0125; ^a^significant differences compared to A; ^b^significant differences compared to D; Mean ± SD: Mean ± Standard Deviation; A: Floor; B: upper limb instability; C: lower limb instability; D: four limbs instability; LT: lower trapezius; UT: upper trapezius; SAO: serratus anterior of opposite; UTO: upper trapezius of opposite

### Changes in two ratios in men and women between four different support surfaces

There was no significant difference in ratios of right lower trapezius/upper trapezius and opposite serratus anterior/upper trapezius between men and women during the SPT exercise on four different surfaces (*p* > 0.05; Table 3).

**Table 3:** Comparison of LT to UT and SAO to UTO ratios in men and women (n = 20)

		A (M ± SD)	B (M ± SD)	C (M ± SD)	D (M ± SD)	F	*p*
LT/UT	Men	2.24 ± 0.68	2.48 ± 0.75	2.25 ± 0.67	2.35 ± 0.70	0.248	0.862
	Women	2.13 ± 0.66	2.37 ± 0.75	2.23 ± 0.74	2.40 ± 0.81	0.327	0.806
	t	0.372	0.336	0.084	0.039		
	P’	0.714	0.741	0.934	0.909		
SAO/UTO	Men	1.26 ± 0.34	1.37 ± 0.35	1.29 ± 0.41	1.39 ± 0.39	0.187	0.904
	Women	1.30 ± 0.34	1.47 ± 0.31	1.31 ± 0.35	1.47 ± 0.28	0.893	0.453
	t	-0.250	-0.645	-0.112	-0.461		
	P’	0.805	0.527	0.912	0.650		

*p < .05 **p’ < 0.0125; Mean ± SD: Mean ± Standard Deviation; Mean ± SD: Mean ± Standard Deviation; A: Floor; B: upper limb instability; C: lower limb instability; D: four limbs instability; LT: lower trapezius; UT: upper trapezius; SAO: serratus anterior of opposite; UTO: upper trapezius of opposite

## Discussion

This study investigated the EMG activities in the lower trapezius muscle during SPT exercise on four different surfaces. De Oliveira et al. reported that the EMG activity of the studied muscles during exercises on a fixed boundary with an external axial load is different according to the base of support used, whether it is a stable or non-stable support [26]. To the best of our knowledge, e this is the first study to assess the activity of the lower trapezius muscle during SPT exercise on four different surfaces.

Our findings demonstrated that the changes in EMG activities of the serratus anterior and lower trapezius muscles during SPT exercise on the upper body unstable surface and whole-body unstable surface were significantly greater than on the other two surfaces (*p* < 0.05). Park et al. [27] reported that the serratus anterior muscle showed greater muscle activity for stabilizing the scapular position on an unstable surface than on a stable surface, while other studies reported that performing push-up exercise on a shaky surface requires greater effort for shoulder and trunk stabilization; especially, serratus anterior muscle activity was highest in the unstable conditions [28, 29]. Our study showed that the anterior serratus and inferior trapezius play a major role in SPT exercise, not just the trapezius.

In this study, according to gender, we found that muscle activities of the opposite upper trapezius and serratus anterior muscles in women were significantly increased on the whole-body unstable surface. We analyzed that this phenomenon is related to the difference in the diameter of female and male muscle fibers. Because men and women's sensorimotor control systems may differ [14], women showed greater activation of the synergist muscles than men to maintain joint instability and facilitate motor control when instability occurs during an isometric shoulder task [10, 30]. Jang et al. assessed the impacts of augmented trunk stabilization with external compression support on the EMG activity of shoulder and scapular muscles and shoulder abductor strength during isometric shoulder abduction [21]. They reported that lower trapezius muscle activity was greater when performing isometric shoulder abduction with no external support than performing with pelvic and thoracic supports [21]. They concluded that augmented trunk stabilization with the external compression support might be advantageous because of reducing the compensatory muscle effort of the upper trapezius during isometric shoulder abduction and increasing shoulder abductor strength. In addition, this study also compared the bilateral upper trapezius muscles and the ratios of two pairs of muscles on both sides, and the result between men and women for the four different surfaces revealed no significant differences (*p* > 0.05). The ratios on both sides exhibited no significant difference that could be attributed to the relatively consistent changes in each muscle as they serve as scapular and glenohumeral stabilizers, respectively [31]. The findings highlighted the importance of trunk stabilization as a component of force production as well as the importance of proximal stability in shoulder and scapular motions during limb movement [32]. Therefore, we designed different unstable surfaces using aircushion balance balls to select the surface that is most effective in activating the target muscle and thus improving the RSP.

Meanwhile, reports of conflicting findings may be due to differences in the tasks performed between MVIC tests and electrode placement [30–33]. Anders et al. [30] suggested that women produced more coactivation of stabilizing muscles during most of the studied isometric contractions [34]. However, such sex differences were not observed in the current study. Differences between those results and ours could reflect the task-specificity of the sex differences in muscle coactivation patterns and/or may be related to the metrics used in each study [35, 36].

## Data Availability

The datasets used and analyzed during the current study are available from the corresponding author upon the request.
